# Linking cognitive flexibility to entrepreneurial alertness and entrepreneurial intention among medical students with the moderating role of entrepreneurial self-efficacy: A second-order moderated mediation model

**DOI:** 10.1371/journal.pone.0256420

**Published:** 2021-09-24

**Authors:** Wang Jiatong, Majid Murad, Cai Li, Shabeeb Ahmad Gill, Sheikh Farhan Ashraf

**Affiliations:** 1 College of International Business, Zhejiang Yuexiu University of Foreign Langugages, Zhejiang, Shaoxing, China; 2 School of Management, Jiangsu University, Zhenjiang, China; 3 Lyallpur Business School, Government College University, Faisalabad, Pakistan; University of Macau, MACAO

## Abstract

This study extended the research on the association between cognitive flexibility and entrepreneurial intention by developing a moderated mediation model. This research examined whether entrepreneurial alertness mediates this association. This study also investigated whether entrepreneurial self-efficacy moderates this mediation model by conducting a moderated mediation model. The sample of this study comprised 486 medical university students of Pakistan. Data gathered using a self-report administered questionnaire and hypotheses were tested with SEM structural equation modeling technique through AMOS user-defined estimates and developed a syntax based on Hayes model 15 of process macro. The results revealed that cognitive flexibility is positively related to entrepreneurial alertness and entrepreneurial intentions. Furthermore, findings showed that the indirect relationship of entrepreneurial alertness via entrepreneurial self-efficacy on cognitive flexibility and the entrepreneurial intention was also significant. This study contributes to the emerging research on psychology and entrepreneurship as well as concludes that individuals with a high level of cognitive flexibility, entrepreneurial alertness, and entrepreneurial self-efficacy are more inclined to pursue a career in entrepreneurship.

## Introduction

Entrepreneurship is an essential driver of societal health and wealth, as well as a formidable engine of economic growth [1]. In fact, with growing unemployment around the globe, many governments are depending on entrepreneurial start-ups for job creation. Previous research over the years focused on understanding the drivers of entrepreneurship by investigating why individuals develop entrepreneurial intentions to become an entrepreneur [2]. Prior studies argued that intentions are assured and considered the best predictor for measuring entrepreneurial behavior [3, 4]. Several authors examined positive personality traits [5] and negative personality traits [6] to predict entrepreneurial intention. Moreover, existing studies identified the importance of the human cognitive perspective that helps in developing their entrepreneurial behavior to start a new business [5–7]. The importance of cognitive view and complexity of human cognition in the field of entrepreneurship is less explored, and there is a need to extend the literature [8, 9]. Cognitive flexibility refers to “*a person’s awareness that in any situation there are many options are alternatives available*, *a willingness to be flexible and adapt to the situation and self-efficacy in being flexible"* [10] p. 625). Research in cognitive psychology explains that an individual can rightly apply knowledge to accomplish a task and make an effective decision that helps introduce solutions to uncertain issues [8, 11, 12].

Foo, Uy [13] explained that cognitive flexibility enables the growth of productive schemas and the identification process of opportunity recognition and exploitation. Miller, Grimes [14] remarked that cognitive flexibility helps individuals to form innovative ideas and possible solutions in difficult situations. Cognitive flexibility empowers individuals to move on to different cognitive styles that motivate them in decision-making even in uncertain and complex environmental situations [15, 16]. Therefore, individuals with a high level of cognitive flexibility are more likely to overcome uncertain problems in different ways and form business start-up activities [17, 18]. Researchers explained that cognitive flexibility is helping individuals to enhance creativity, problem-solving, identification of opportunity recognition, and exploitation [8, 19, 20]. Thus, in the formation of the entrepreneurial start-up process, these abilities improve individuals’ mindset level and control uncertain problems through a cognitive perspective [21, 22].

In short, we seek to contribute to the literature of cognitive psychology and entrepreneurship in different aspects. First, we extend the literature of prior researchers by contributing to cognitive flexibility that impacts an individual’s perceived fit and attitude to become an entrepreneur [8, 23, 24]. There is less empirical research available on cognitive flexibility, entrepreneurial alertness, and entrepreneurial intention. A recent study by Dheer and Lenartowicz [8], suggested that research is more needed in the field of cognitive psychology and entrepreneurship. The cognitive mindset of an individual to starting a new business is still an open-ended question in research that why some individuals have entrepreneurial intentions to become entrepreneurs rather than others. To address this question, this study aims to identify the influence of cognitive flexibility on entrepreneurial intention. Second, this study contributes to the literature on the mediating role of entrepreneurial alertness in the relationship between cognitive flexibility and entrepreneurial intentions [8]. Previous studies explored entrepreneurial alertness as a predictor and outcome variable to examine entrepreneurial intention [25–27]; the mediation role of alertness on intention was less studied in the existing literature [28, 29]. Therefore, to address this gap, we advance knowledge regarding entrepreneurial alertness toward cognitive flexibility and entrepreneurial intention that why individuals are more alert to identify an opportunity for starting a new business.

Third, most of the prior researches focused on the indirect, direct influence of entrepreneurial self-efficacy on different entrepreneurial outcomes, such as intention, orientation, alertness, and entrepreneurial success [30–32]. As suggested by [33, 34], entrepreneurial self-efficacy can take a mediator and moderator in the association of entrepreneurial intentions to entrepreneurial actions. A recent study has not shown an interest in exploring the moderating influence of entrepreneurial self-efficacy [8]. Thus, to fill this gap, this study predicts that entrepreneurial self-efficacy strengthens the positive effect of cognitive flexibility and entrepreneurial alertness on entrepreneurial intention. Fourth, this study discussed the person-environment fit theory [35] and theory of planned behavior [36] for adding a theoretical contribution to identify the entrepreneurial intention to escalate the scope of these theories that how cognitive attributes can improve the understanding among individuals to start a new business venture.

### Theory and hypotheses development

Person-environment fit theory is proposed by [35]. This theory focuses on the interaction between characteristics of the individual and environment, whereby the individual not only influences his or her environment, but the environment also affects the individual [37]. The importance of this theory is underlying in different perspectives. It suggests that individuals’ needs and actions are different. It is also explained that the environment is different according to their norm, values, and expectations. A study remarked that individuals who are more inclined toward the environment would evaluate their skills and abilities according to the environmental situations [38]. Furthermore, the person-environment fit theory was widely discussed by prior researchers in the domain of human resource management [39], organizational behavior [38], and entrepreneurship [37]. Markman and Baron [40] found that explaining individuals’ perceived fit as a career in entrepreneurship positively influenced by human and social capital. Prottas [41] suggested that a wish to pursue autonomy might make an individual intending to start a new business.

Additionally, previous literature argued that entrepreneurial intention is related to an individual’s willingness to form entrepreneurial behavior [8, 18, 42, 43]. Based on the theory of planned behavior (TPB) intention is the best predictor to measure entrepreneurial behavior [36]. The TPB views behavioral intentions as the crucial proximal predictor of behavior. The formation of individual behavioral intentions are associated with three variables: ATT attitudes (individuals cognitive and psychological assessment of the extent to which a particular behavior is desirable or not), SN subjective norms (individuals perception toward the social pressure that comes from the friends and family members), and perceived behavioral control PBC (individuals perceived easiness with which a particular behavior can be performed [1]. The TPB explained that there is a positive influence of entrepreneurial intention on entrepreneurial behavior, and further, it has been confirmed by recent literature on entrepreneurial intention/behavior models [3, 4]. Thus, based on the existing studies researchers less explored the role of cognitive flexibility on entrepreneurial alertness, and entrepreneurial intention, therefore, it is a need to the extent this theoretical conceptualization through a person-environment fit and theory of planned behavior in the field of cognitive psychology and entrepreneurship [44–46].

### Cognitive flexibility and entrepreneurial intention

According to Martin and Rubin [10], flexibility refers to a vital communication element to perform a specific task. Prior researchers developed measures for being flexible in social and environmental conditions [47, 48]. However, before individuals can show flexibility, they should first be cognitively flexible and have an awareness of the environment [49, 50]. Cognitive abilities facilitate individuals to perceive opportunities and apply knowledge to pursue a career in entrepreneurship [6, 51, 52]. Drawing from the previous literature, cognitive flexibility enhances the creativity, innovativeness among individuals to consider many perceptions of awareness and bring many solutions to a problem [19, 53, 54]. In the process of cognitive flexibility, an individual must have an understanding of new opportunities to solve social and environmental hurdles [55, 56]. From the perspective of the neurological view, cognitive flexibility is related to reduce the level of self-consciousness and effective functioning of the operational memory [57]. Therefore, individuals with a higher level of cognitive flexibility show can hold information which allows them to create novel ideas and control uncertain environment situations [58, 59].

Furthermore, cognitive flexibility increases the individual opinion to perform entrepreneurial activities [60, 61]. Cognitive flexibility creates more awareness because a high level of cognitive mind individuals is more inclined to show creativity and innovativeness to adapt entrepreneurial ideas [62, 63]. Thus, based on this discussion, we argued that cognitive abilities are the dominant features of entrepreneurship and provide this understanding that individuals with a higher level of creative minds are more likely to become entrepreneurs [64, 65]. Thus, we hypothesized;

**H1:** Cognitive flexibility is positively related to entrepreneurial intention.

### Cognitive flexibility and entrepreneurial alertness

Entrepreneurs may be as different from each other as they are from the rest of the population [66]. This understanding is developed because of existing findings from personality research in particular and created the cognitive aspect of entrepreneurship [54]. A prior study found that entrepreneurs who do not feel to take risks while starting a new business rather than non-entrepreneurs are more likely to control uncertain situations [23, 67]. It is observed that the entrepreneur’s traits do not explain their behaviors until they have appropriate information and cognitive ability [68, 69].

According to the cognitive perspective in entrepreneurial alertness, individuals with cognitive abilities are more prepared for the identification and exploitation of opportunities [54, 63]. Therefore, individuals continually search and see opportunities and ready to exploit them according to their cognitive levels [66]. Furthermore, existing studies explained that some individuals are alert, and some are not [63, 68]. Individuals who are more alert have new knowledge of the market, higher level of intelligence, which encourages them to start new ventures [69, 70]. Thus, it is seen that entrepreneurial alertness built cognitive abilities among individuals and develops social, environmental conditions that generate innovative solutions to business startups [9, 26, 71]. Therefore, it is possible to discuss that entrepreneurs who have cognitive traits can see business opportunities better than non-entrepreneurs who do not have cognitive abilities. Hence, the proposed hypothesis is predicted;

**H2:** Cognitive flexibility is positively related to entrepreneurial alertness.

### Entrepreneurial alertness and entrepreneurial intention

Entrepreneurial intention helps individuals in shaping their entrepreneurial behaviors to start a new business [2]. The relationship between entrepreneurial alertness and entrepreneurial intention has been defined by previous researchers [4, 25, 29, 68]. Most of the studies found that entrepreneurial alertness had a positive and significant influence on entrepreneurial intention [26, 28, 72, 73]. Entrepreneurial intention refers to a conscious state of mind that precedes action toward entrepreneurial behaviors [74, 75]. Moreover, the entrepreneurial intention is associated with the self-acknowledged belief by an individual that they aim to start a new business and intentionally plan to do so in the future [76].

Furthermore, entrepreneurial alertness is defined as a cognitive ability that positively affects both opportunity identification and exploitation that contains opinion, pattern recognition, and evaluation [77]. Tang, Kacmar [68] found that entrepreneurial alertness could be measured through three dimensions; 1) scanning and searching; systematically and non-systematically scan the internal and external environment and gather information, 2) association and information; associate together scanned and searched unconnected information, 3) judgment and evaluation; make judgment and evaluation according to the commercialize ability of the idea to pursue new business. Thus, individuals with a higher level of these dimensions are more likely to start new ventures. Accordingly, we predicted the following hypothesis;

**H3:** Entrepreneurial alertness is positively related to entrepreneurial intention.

### Moderated-mediation of entrepreneurial self-efficacy

Entrepreneurial self-efficacy refers to an individual’s beliefs in his/her ability to accomplish tasks and roles aimed at entrepreneurial start-ups [78]. Many researchers discussed the importance of entrepreneurial self-efficacy in the area of entrepreneurship because of its direct and indirect role in identifying entrepreneurial intentions, opportunity recognition, and organization performance [58, 79–82]. According to Bandura [83], entrepreneurial self-efficacy states to *“a cognitively created motivation*.*”* The literature on self-efficacy and cognitive flexibility has been studied by a few researchers in the past. Studies on entrepreneurial self-efficacy examined the role of gender [84] experience and education [85] among its cognitive factors [8].

Previous literature suggested the concept of entrepreneurial self-efficacy associated with the individual belief and ability to perform the task for developing new business ventures [86–88]. Furthermore, McGee and Peterson [82] stated that entrepreneurial self-efficacy has a positive direct effect on entrepreneurial outcomes and new venture performance. Dheer and Lenartowicz [8] found that individuals’ belief in self-efficacy is a powerful resilient force for the implementation of their entrepreneurial intention.

Hmieleski and Corbett [89] found that entrepreneurial self-efficacy positively moderated by the relationship between entrepreneurial behavior and venture performance. Thus, individuals with a high level of entrepreneurial self-efficacy are more likely to be alert to recognize new opportunities, where such alert individuals are more likely to chase opportunities frequently, which in turn enhance their levels of entrepreneurial self-efficacy [90]. Chen, Yang [91] investigated a study on 366 individuals’ role of cognitive flexibility on creativity and innovativeness. The findings indicate that cognitive flexibility helps individuals to make novel ideas and promotion of innovative discoveries. Cognitive flexibility is related to creativity, innovativeness, and creative thinking that empower individuals to come up with innovative thoughts and solutions to problems that may affect the formation of new businesses [19, 92].

The role of the social cognitive theory facilitates the individual’s beliefs and develops a high level of self-efficacy toward engaging in the new business formation process [93, 94]. Considering prior research in entrepreneurship [8] found that entrepreneurial self-efficacy mediates in the relationship between cognitive flexibility and entrepreneurial intention. This objective is to explore and extend the previous literature using entrepreneurial self-efficacy as a moderator and mediator in the relationship between cognitive flexibility, entrepreneurial alertness, and entrepreneurial intention or not. Therefore, we hypothesized the following hypotheses;

**H4:** Entrepreneurial self-efficacy moderates the relationship between cognitive flexibility and entrepreneurial intention. Specifically, the positive relationship between cognitive flexibility and entrepreneurial intentions will be weaker (stronger) when entrepreneurial self-efficacy is high (low).
**H5:** Entrepreneurial self-efficacy moderates the relationship between entrepreneurial alertness and entrepreneurial intention. Specifically, the positive relationship between entrepreneurial alertness and entrepreneurial intentions will be weaker (stronger) when entrepreneurial self-efficacy is high (low).

**Fig 1:**
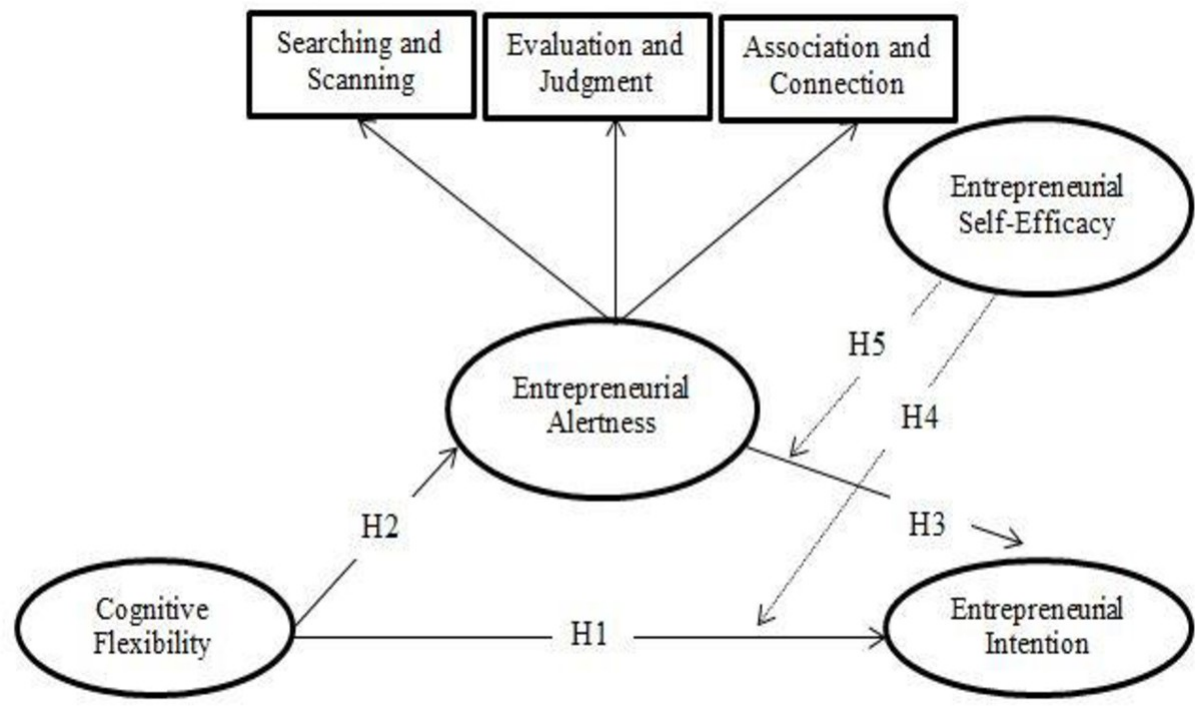
Conceptual model.

The research model is depicted in Fig 1. The model explains that cogitative flexibility positively predicts entrepreneurial alertness that, in turn, positively predicts entrepreneurial intentions, as well as that the positive impacts of cognitive flexibility and entrepreneurial alertness on entrepreneurial intention vary across the values of the entrepreneurial self-efficacy.

## Research methodology

### Population and sampling techniques

The nature of the study was cross-sectional and based on the primary data. The targeted population included medical students of ten public and private sector universities of province Punjab, Pakistan. This study gathered data from the province of Punjab because Punjab is the biggest province rather than other provinces of Pakistan population-wise, and students from all over Pakistan migrate there for the completion of their studies. The total population of the selected students was approximately 4000 who are practicing house jobs and have entrepreneurial intentions to start their own medical clinics. The previous researcher argued that students are appropriate samples when the study is focused on the prediction of entrepreneurial intention or behavior because generally, students form their intentions to start business development activities in their earlier stages of academic degrees [4, 58, 90, 95].

According to [96], if the target population is more than 4000, a minimum sample size of 500 is enough. Moreover, a non-probability (convenience sampling technique) was applied to select respondents from universities in the province of Punjab, Pakistan. As suggested by [97] the formula for calculating the sample size Z^2^ * p(1 –p)/e^2^, where z = 1.6384, p = 0.25, and e^2^ = 0.0016, the sample size for this study is approximately 350. Furthermore, to collect more responses, we distributed 600 paper-and-pencil questionnaires among the students during their free time. The data was gathered from January 2020 to April 2020. The original draft of the questionnaire was in English because English is the official teaching language in secondary and higher education institutions of Pakistan. Considering the Podsakoff, MacKenzie [98] approach to reduce the possibility of common method bias (CMB), the participation of the students was voluntary, and confidentially of their responses was assured. A total of 510 questionnaires were returned by the participants with a participation rate of 85%. Some of the questionnaires, around 24, were discarded due to incomplete forms of filling. Thus, the final sample size was 486 participants and further used for analysis.

#### Profile of the respondents

We selected ten public and private sector medical university students for data collection. The list of the targeted universities was presented in Table 1 and SI Appendix is presenting the details of measurement items. The demographic information of the participants was 56.7% male and 43.4% female. The majority of the participants were between the ages of 18–25 years. Moreover, 43% of participates came from entrepreneurial family backgrounds.

**Table 1:** List of medical universities.

Sr. No	University Name	Questionnaire Distributed	Collected Questionnaire
1	King Edward Medical University, Lahore	100	82
2	Nishter Medical University, Multan	100	86
3	Punjab Medical University, Faisalabad	50	43
4	Federal Medical And Dental College, Islamabad	50	42
5	Sargodha Medical College, Sargodha	50	39
6	Allama Iqbal Medical College, Lahore	50	45
7	University Medical And Dental College, Faisalabad	50	43
8	Lahore Medical And Dental College, Lahore	50	44
9	Gulab Devi Medical College, Lahore	50	40
10	Independent Medical College, Faisalabad	50	46
		Total = 600	Total = 510

### Instruments

#### Cognitive flexibility

To assess cognitive flexibility, we adapted twelve measurement items from the study of [10] using a five-point Likert scale. This scale was used and verified by previous researchers [8]. A sample item, “I can communicate an idea in many different ways” (Cronbach’s α = 0.947).

#### Entrepreneurial alertness

To measure entrepreneurial alertness, we adapted thirteen items scale validated by [68] using a five-point Likert scale. This scale was used by many researchers in prior studies [1, 23, 28]. The scale has a further three dimensions; first, searching and scanning have six measurement constructs. A sample item, "I am always actively looking for new information” (Cronbach’s α = 0.941). Second, association and connection have three items. A sample item, "I am good at connecting dots" (Cronbach’s α = 0.915). Third, evaluation and judgment have four constructs. A sample item, "I have a gut feeling for potential opportunities" (Cronbach’s α = 0.935). We validate these dimensions using second-order CFA analysis to identify the total variance of these factors. We found 72% variance with each of the three dimensions accounting for 21%, 25%, and 26%.

#### Entrepreneurial intention

To assess entrepreneurial intention, we adapted six items using a five-point Likert scale [99]. This scale was widely used by previous researchers to measure entrepreneurial intention [4, 100]. A sample item, "I am ready to do anything to be an entrepreneur” (Cronbach’s α = 0.945).

#### Entrepreneurial self-efficacy

To measure entrepreneurial self-efficacy, we adapted four measurement items on a five-point Likert scale [80]. A sample item "I can convince that I can think creatively" (Cronbach’s α = 0.941).

### Ethical statement

This study was carried out in accordance with the recommendations of Ethical Principles of Psychologists and Code of Conduct by the American Psychological Association (APA). All participants gave written informed consent in accordance with the Declaration of Helsinki. The protocol was approved by the ethics committee of Lyallpur Business School, Government College University, Faisalabad, Pakistan.

## Results

### Measurement of model

**Fig 2:**
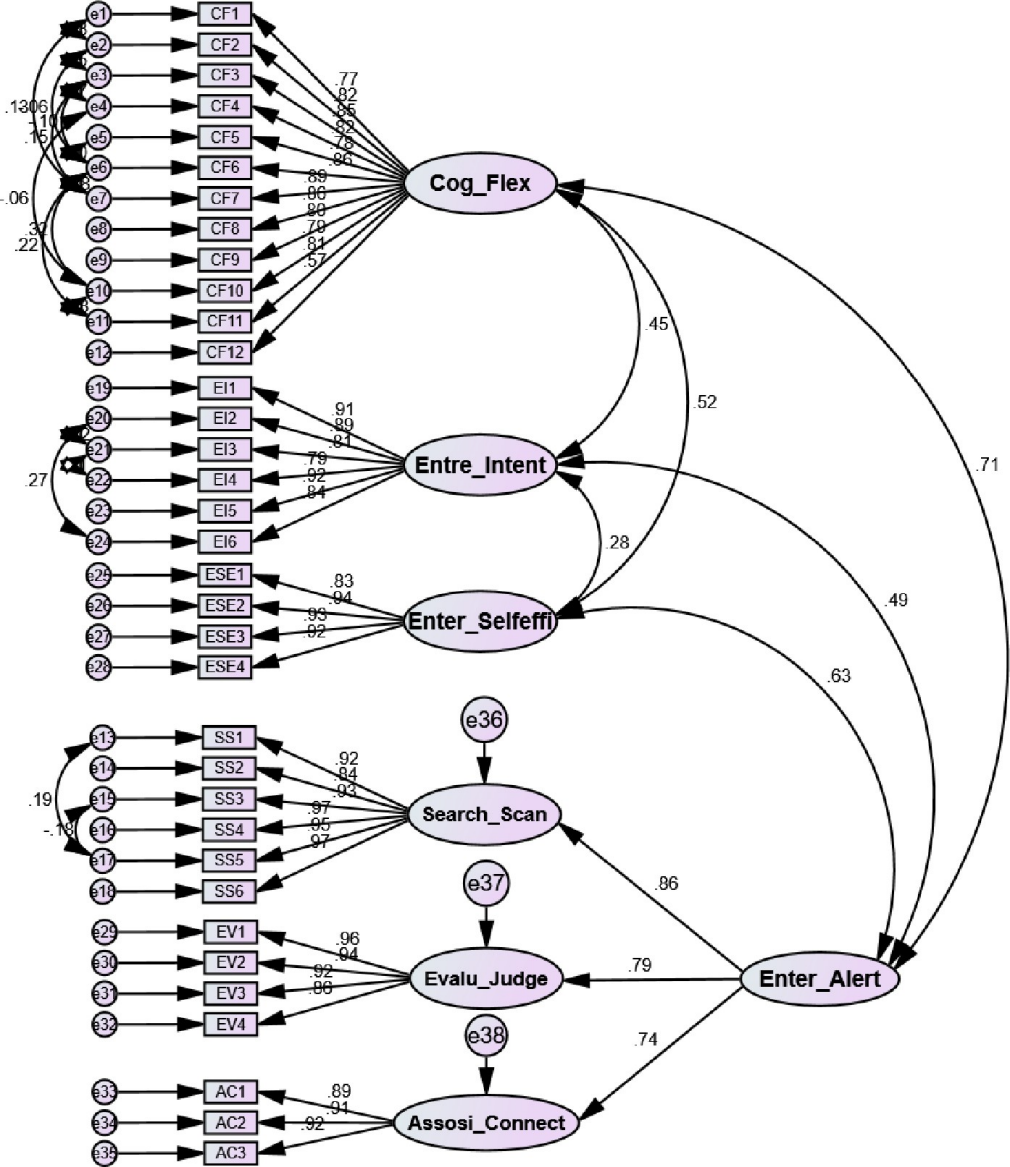
Measurement model.

To check the fitness of the measurement model, we performed exploratory factor analysis EFA and confirmatory factor analysis CFA in SPSS and AMOS software. The results of EFA and CFA were presented in Table 2 and Fig 2. For the goodness-of-fit the results were stated as follows: X^2^ = 1604.354, df = 545, X^2^/df = 2.944<5, CFI = 0.932, TLI = 0.926, IFI = 0.932, RFI = 0.892, NFI = 0.901, GFI = 0.835, AGFI = 0.809, RMR = 0.033, SRMR = 0.043 and RMSEA = 0.060. Thus, all the constructs meet the criteria for the measurement model [101]. The square multiple correlation values were also satisfactory following the threshold above 0.3 was acceptable and above 0.50 considered as ideal.

**Table 2:** Factor analysis for the measurement model.

Second-order Factors	First-order Factors	Items	1	2	3	4	5	6	CR	AVE	Communalities
Entrepreneurial Alertness	Searching and Scanning	SS1	.797						.948	.731	.724
		SS2	.758								.647
		SS3	.817								.769
		SS4	.875								.873
		SS5	.872								.845
		SS6	.888								.878
	Association and Connection	AC1		.790					.916	.783	.821
		AC2		.862							.859
		AC3		.861							.872
	Evaluation and Judgment	EV1			.850				.946	.789	.893
		EV2			.852						.877
		EV3			.837						.845
		EV4			.837						.797
	Entrepreneurial Intention	EI1				.893			.960	.749	.830
		EI2				.867					.817
		EI3				.809					.720
		EI4				.793					.699
		EI5				.898					.846
		EI6				.846					.774
	Entrepreneurial Self-Efficacy	ESE1					.805		.952	.803	.752
		ESE2					.890				.882
		ESE3					.876				.866
		ESE4					.891				.864
	Cognitive Flexibility	CF1						.739	953	603	.603
		CF2						.788			.689
		CF3						.791			.708
		CF4						.758			.655
		CF5						.727			.607
		CF6						.777			.753
		CF7						.837			.769
		CF8						.816			.720
		CF9						.745			.620
		CF10						.758			.635
		CF11						.792			.674
		CF12						.533			.377
	**Eigenvalue**		**3.81**	**1.44**	**2.23**	**3.61**	**2.51**	**12.9**			

### Reliability and validity analysis

Constructs composite reliability and convergent validity were assessed through the master validity analysis. The results of the composite reliability values were ranged from 0.916 to 0.960 exceeded the proposed benchmark of 0.70 [102]. Moreover, convergent validity was evaluated using the average variance extracted (AVE). In Table 2, it is observed that the AVE values were ranged from 0.603 to 0.803, which exceeded the acceptable level of the convergent validity threshold value of 0.50 [103].

### Discriminant validity, descriptive statistics, and correlations

Discriminant validity was assessed using the criteria of [103]. Table 3 indicates that values with diagonals are the square root of the AVE is discriminant validity, and values under diagonals are correlations between variables. We found that there was a positive and significant correlation between cognitive flexibility and entrepreneurial intention (r = 0.408, *p* = 0.01). Moreover, it was positive and significant correlations of scanning and searching (r = 0.364, *p* = 0.01), association and connection (r = 0.426, *p* = 0.01), evaluation and judgment (r = 0.416, *p* = 0.01) and entrepreneurial self-efficacy (r = 0.442, *p* = 0.01) with entrepreneurial intention.

**Table 3:** Discriminant validity and correlation.

	M	S.D	CF	EI	SS	EV	ESE	AC
CF	3.76	0.711	**0.777**					
EI	3.77	0.747	0.408***	**0.865**				
SS	3.94	0.774	0.364***	0.353***	**0.855**			
EV	3.51	0.804	0.416***	0.354***	0.177***	**0.888**		
ESE	3.75	0.889	0.442***	0.426***	0.222***	0.443***	**0.896**	
AC	3.71	0.872	0.426***	0.412***	0.226***	0.296***	0.540***	**0.885**

**Note:** CF = Cognitive flexibility, SS = Scanning, and Search, AC = Association and connection, EV = Evaluation and judgment, ESE = Entrepreneurial self-efficacy, EI = Entrepreneurial Intention are predictors.

Values with diagonals are the square root of the AVE

Values under diagonals are correlations ***significant (*p<0*.*001)*

### Common method bias

We applied two methods to check the possibility of common method bias in the data. First, Harman’s single factor test was performed using SPSS software, according to Harman’s methodology, common method bias is present when one factor emerges from factor analysis and explains >50% of the variance. We moved all the items into a one-factor analysis using the rotated solution and found six factors; with the first factor, eigenvalue explains 36.94% of the variance <50%. However, this method is an outdated approach nowadays and not used due to its limitations [104]. Second, the latent factor test is recommended by Podsakoff, MacKenzie [98]. Therefore, we used the latent factor and calculated the standardized regression weights by including them in the measurement model, and then we calculated the standardized regression weights after excluding them from the measurement model in AMOS. However, the difference between the two situations was below the threshold value of (delta>0.2). Thus, it is evident that there is not an issue of common method bias in this study.

### Data analysis method

Before going to analyze the structural model, we checked the issue of multicollinearity by examining the variance inflation factor. The findings show that cognitive flexibility, VIF was 1.482, entrepreneurial alertness VIF was 1.622, and entrepreneurial self-efficacy, VIF was 1.395. All the values of VIF were below the threshold value of 10 recommended by [105]. To test the direct, indirect, and moderating relationships among variables, there are several methods used by previous researchers [106, 107]. In this conceptual model, we analyzed the direct effect of cognitive flexibility on entrepreneurial intention and examined the direct effect of entrepreneurial alertness on cognitive flexibility as well as the direct effect of entrepreneurial alertness on entrepreneurial intention. Furthermore, we analyzed the moderated mediation effects of entrepreneurial self-efficacy in the relationship between cognitive flexibility, entrepreneurial alertness, and entrepreneurial intention. For the prediction of indirect effect, some studies [29, 104] used the [106] approach. Similarly, some authors Preacher, Rucker [107] criticized this approach and suggested that this test did not provide robust statistical power and it is also cannot offer the combined analysis of direct and indirect effects and accurate estimation of the indirect result of the predictor on the criterion variable [108]. Therefore, several studies recommended that SEM structural equation modeling is the best tool for predicting direct, indirect, and moderating effects combined in the structural model because it provides more robust results as compared to traditional data analysis methods [2, 3, 23].

### Structural model

We applied the structural equation modeling (SEM) technique to test the hypotheses. The significance of this model, it is a second-order structural model, and all the items are estimating the complete model in a single run through AMOS software. During (SEM) it is most important to provide all the measures with their contribution to the structural model. Meanwhile, we tested the second-order structural model in a one-click, which has no evidence in past studies we explored, but to justify the statistical approach, we distributed it in a process macro. We explained all the study hypotheses from 1 to 5 regarding the second-order moderated mediation for testing the direct and indirect relationships, which was statistically more similar to model 15 presented in process macro by [109]. In that model 15 Hayes [109] explained the procedure for testing the second-order moderated mediation of a single mediator and moderator for direct and indirect effects in a unique, robust model. However, the AMOS has not the built-in capacity to run model 15 in one click, like SPSS. However, through a well-constructed user-defined estimate (i.e., machine language called syntax), AMOS becomes able to run this model in one click.

The following equations were used in AMOS syntax to run the direct and indirect paths for second-order moderated mediation:

1. Indirect path = A*(B1+(B2*V))
2. Direct path = C1+(C3*V)

**Fig 3:**
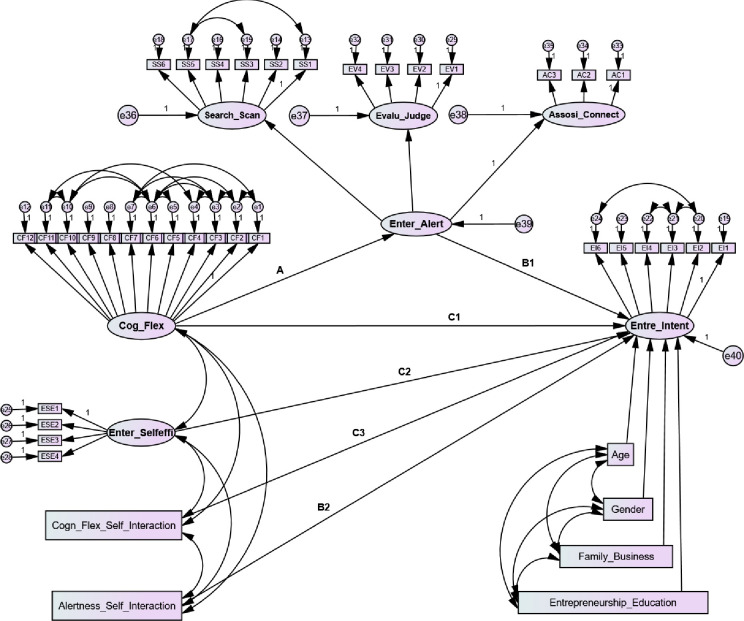
Under-defined path model.

To follow these equations, we estimated entrepreneurial self-efficacy at high, medium, and low levels for conditional direct and indirect effects defined in the model. To fulfill the assumptions of the user-defined estimate to run a statistically robust model, we analyzed it by using 5000 bootstrapped and at a 95% confidence interval. The possible understanding of user-defined estimates in syntax for AMOS shows in Fig 3. To more specifically test the conditional direct and indirect moderated mediation of entrepreneurial self-efficacy was estimated on high (+1sd), medium, and below (-1sd).

### Hypothesis testing

**Fig 4:**
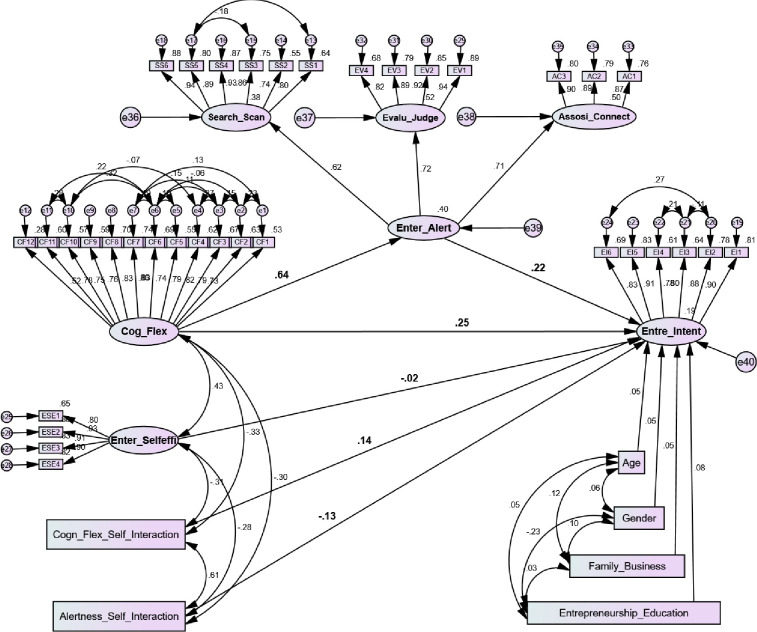
Structural model.

To assess the structural model R^2^, we found that the structural model explained a 40% variance in entrepreneurial alertness and a 19% variance in entrepreneurial intention. As suggested by Chin [110], desired R^2^ values should be greater than 0.1 or zero. Therefore, it is not surprising as most of the entrepreneurial intention and behavior-based studies have explained a 20% to 40% variance in their prior studies [4, 58, 95]. Furthermore, we tested the hypothesis relationships. The findings of the hypotheses are expressed with standardized estimates, critical ratios, and *p* values. Table 4 and Fig 4 presented the results. As hypothesized in the model, H1 cognitive flexibility is positively related to entrepreneurial intention. The findings indicate that cognitive flexibility has a direct positive and significant impact on entrepreneurial intention (β = 0.299, t = 3.44, *p*<0.000). Thus, H1 was supported. This relationship stated that individuals with a higher level of cognitive flexibility have more awareness and decision-making power to pursue a career in entrepreneurship.

**Table 4:** Direct, indirect & conditional effects.

Hypothesis & Paths			β	t-Value	P	Bias-corrected Percentile 95% CI				Label
						Estimate	Lower	Upper	P	
CF	→	EI	.299	3.443	***	.247	.097	.407	.003	**C1**
CF	→	EA	.573	9.646	***	.636	.555	.711	.000	**A**
EA	→	EI	.294	2.909	.004	.219	.033	.411	.018	**B1**
CF x ESE	→	EI	.092	2.515	.012	.140	.009	.271	.037	**C3**
EA x ESE	→	EI	-.076	-2.33	.020	-.127	-.272	.019	.084	**B2**
*Controls*										
Entrepreneurial Education	→	EI	.146	1.809	.070	.080	-.013	.169	.106	
Family Business	→	EI	.072	1.075	.282	.046	-.040	.132	.299	
Gender	→	EI	.072	1.046	.295	.046	-.040	.133	.317	
Age	→	EI	.041	1.135	.257	.049	-.039	.131	.266	

**Note:** CF = Cognitive flexibility; EA = Entrepreneurial alertness; ESE = Entrepreneurial self-efficacy; EI = Entrepreneurial Intention; β = Standardized Coefficient Estimates; SE = Standard Error; *p* = level of significance; Label = Syntax; Bootstrapping = 5000; CI = confidence of interval 95%

(*p<0.05; **p<0.*01;*

***p<0.*001)*

Moreover, we tested H2 cognitive flexibility positively related to entrepreneurial alertness. The results show that cognitive flexibility has a direct positive and significant influence on entrepreneurial alertness (β = 0.573, t = 9.64, *p*<0.000). Therefore, H2 was supported. Individuals who have cognitive abilities are more inclined to identify and recognize the opportunities for starting a new business venture. Furthermore, we hypothesized H3 entrepreneurial alertness positively related to entrepreneurial intention. The results illustrate that entrepreneurial alertness has a direct positive and significant effect on entrepreneurial intention (β = 0.294, t = 2.90, *p*<0.004). Consequently, H3 was accepted. This association indicated that individuals with a higher level of alertness through scanning and search, association and connection, evaluation, and judgment are more actively recognize and exploit new opportunities. Additionally, we tested H4 entrepreneurial self-efficacy moderates the strength of the direct relationship between cognitive flexibility and entrepreneurial intention in the way that the relationship will be stronger for the individuals who are higher in entrepreneurial self-efficacy than for those who are lower in entrepreneurial self-efficacy. According to Hayes [109] procedure of user-defined estimate explained in model 15, Table 4 findings show that entrepreneurial self-efficacy significantly moderates the direct relationship between cognitive flexibility and entrepreneurial intentions (β = 0.092, t = 2.51, *p*<0.012), Thus, H4 was accepted. Meanwhile, we tested H5 entrepreneurial self-efficacy moderates the mediated relationship between cognitive flexibility and entrepreneurial intention by entrepreneurial alertness, in the way that the mediated relationship will be stronger for those who are higher in entrepreneurial self-efficacy. According to Hayes [109] procedure, the results of user-defined estimates through AMOS syntax shows in Table 4, which indicated that entrepreneurial alertness and entrepreneurial self-efficacy have a negative but significant moderated mediation effect on entrepreneurial intention (β = -0.076, t = -2.30, *p*<0.020). Hence, H5 confirmed partial moderated mediation and accepted.

#### Testing the conditional direct and indirect effects

To test the moderated mediation model relationship [109] suggested four conditions without attaining this moderated mediation do not exist. The suggestions are following, a) the relationship between exogenous and endogenous should significant; b) the interaction of moderator and mediator on endogenous should significant; c) the relationship between the mediator and the endogenous variable should be significant; d) the degree of conditional indirect effect has to be different at low, medium and high levels for moderator.

**Fig 5:**
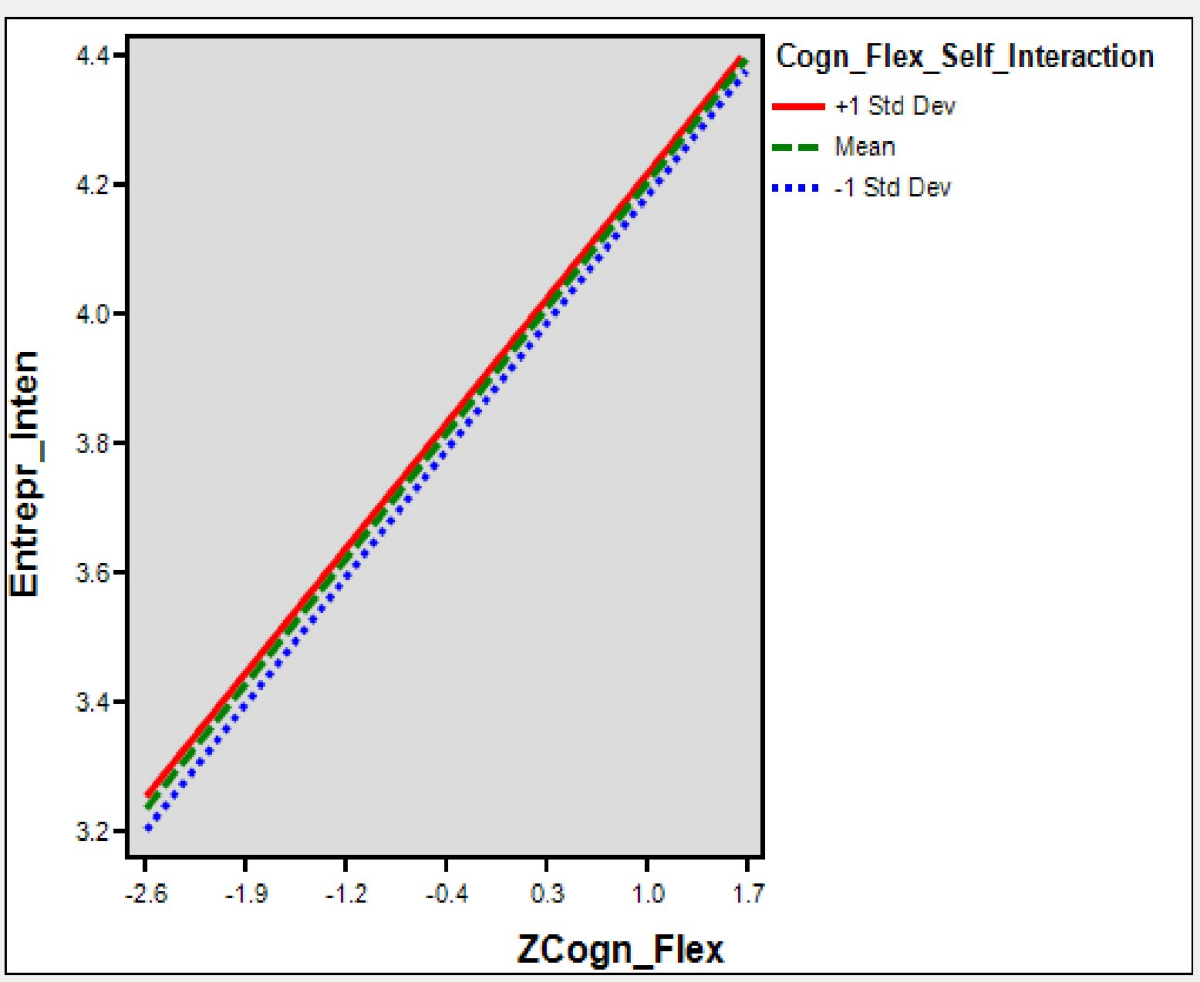
Interaction ESE-CF and EI.

To test the conditional direct effect through H4, we found a significant relationship between cognitive flexibility and entrepreneurial intention (β = 0.299, t = 3.44, *p*<0.001). The interaction effect between cognitive flexibility and entrepreneurial self-efficacy is also significant that confirms a moderating influence (β = 0.092, t = 2.51, *p*<0.012). Furthermore, Table 5 and Fig 5, shows that there is a moderating effect of entrepreneurial self-efficacy in the relationship between cognitive flexibility and entrepreneurial intention. The results indicate that (β = 0.37, *p*<0.001) high levels of entrepreneurial self-efficacy (+1sd) for individuals and (β = 0.21, *p*<0.001) for low levels of entrepreneurial self-efficacy (-1sd).

**Table 5:** Conditional direct and indirect effect of cognitive flexibility on entrepreneurial intention through entrepreneurial self-efficacy.

	Β	Percentile 95% CI		P
		Lower Bound	Upper Bound	
The conditional indirect effect at high, medium and low entrepreneurial self-efficacy				
Low (-1sd) entrepreneurial self-efficacy	.206	.054	.382	***
Medium (0) entrepreneurial self-efficacy	.169	.024	.340	***
High (+1sd) entrepreneurial self-efficacy	.131	-.016	.315	Insignificant
The conditional direct effect at high, medium and low entrepreneurial self-efficacy				
Low (-1sd) entrepreneurial self-efficacy	.219	.009	.435	***
Medium (0) entrepreneurial self-efficacy	.299	.115	.500	***
High (+1sd) entrepreneurial self-efficacy	.379	.187	.586	***

**Note:** Bootstrapping sample size = 5000; β = Standardized estimate

(*p<0.05; **p<0.*01;*

***p<0.*001)*

**Fig 6:**
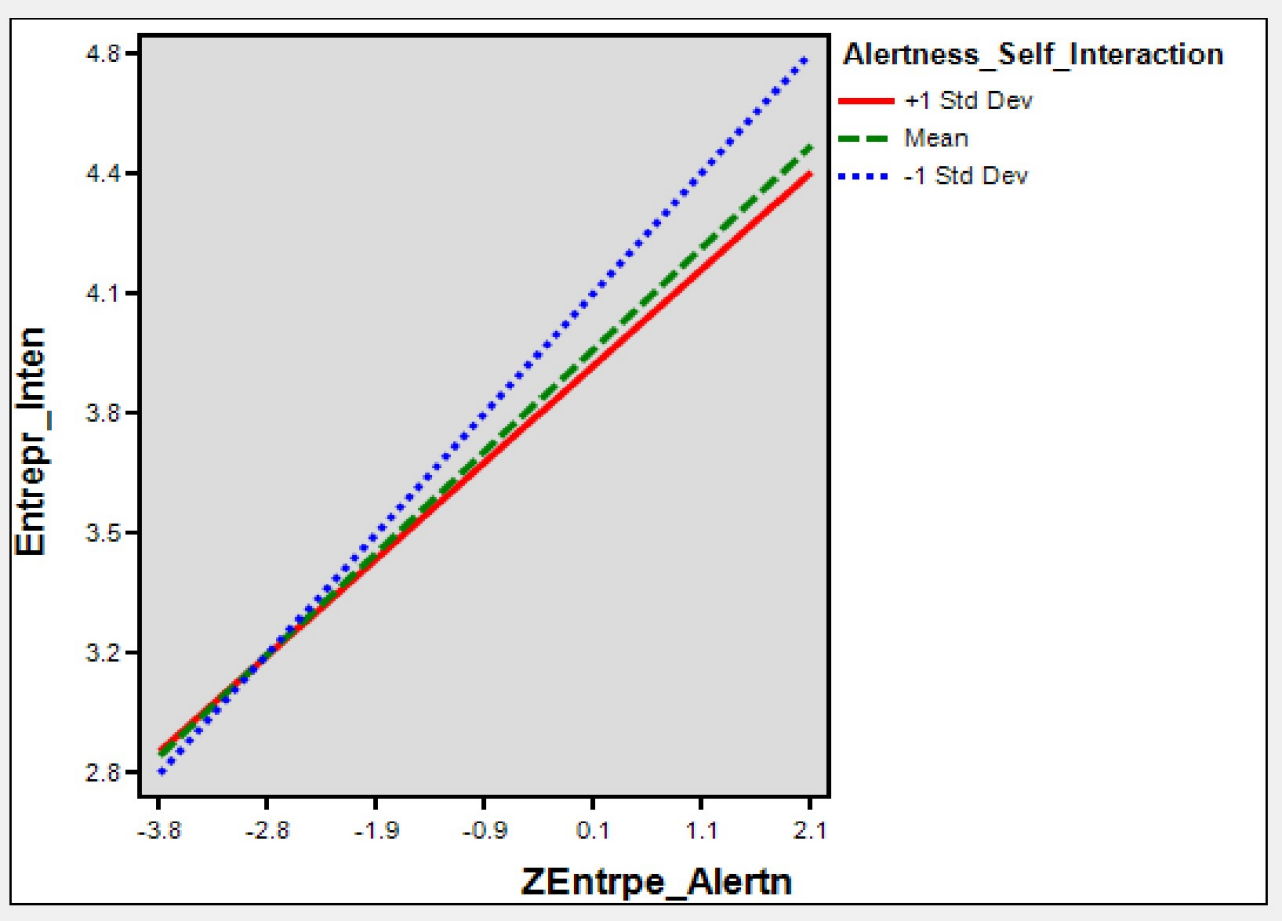
Interaction ESE-EA and EI.

To test the conditional indirect effect through H5, Table 3 shows that (β = 0.299, t = 3.44, *p*<0.001) significant relationship between cognitive flexibility and entrepreneurial intention and met with the condition (a). The interaction effect (β = -0.076, t = -2.3, *p*<0.020) between entrepreneurial alertness and entrepreneurial self-efficacy is also significant that satisfies the condition (b). Table 3 shows that entrepreneurial alertness has a direct positive and significant effect on entrepreneurial intention (β = 0.294, t = 2.90, *p*<0.004) that met the condition criteria (c). Thus, Table 5 and Fig 6 shows that the conditional indirect effect of cognitive flexibility on entrepreneurial intention through entrepreneurial self-efficacy (β = 0.13, *p* = -0.016; 0.315) that is positive but not significant for high levels of entrepreneurial self-efficacy (+1sd) for individuals and conversely (β = 0.206, *p* = -0.054; 0.382) is a positive sign for low levels (-1sd) of individuals. Thus, hypothesis 5 of this study is not consistent.

## Conclusion and discussion

This study highlights the importance of cognitive flexibility on entrepreneurial intention through the mediation role of entrepreneurial alertness and the moderating effect of entrepreneurial self-efficacy. This study added a contribution in the field of cognitive psychology literature to enhance the understanding that individuals with a higher level of cognitive flexibility are more inclined to start a new venture. Moreover, results indicated a positive and significant relationship between cognitive flexibility, entrepreneurial alertness, and entrepreneurial intention. Furthermore, findings show that entrepreneurial self-efficacy strengthens the relationship between cognitive flexibility, entrepreneurial alertness, and entrepreneurial intention.

Concerning H1, we found that cognitive flexibility has a positive and significant impact on entrepreneurial intention. This hypothesis was accepted, and the results are in line with the previous researchers [8, 111, 112]. Laureiro-Martínez, Brusoni [15] found that individuals who perceive higher cognitive abilities have more awareness to handle problematic situations. Besides, our results are similar to [66, 70] who remarked that individuals’ cognitive abilities not only crucial for recognition of opportunities it is also persuading perceptions that individuals can pursue the role and chores of entrepreneurs. Furthermore, studies on cognitive psychology found the positive role of cognitive flexibility for creativity, innovativeness, adaptability, and problem-solving skills [18, 53, 56]; this study contributed these significant findings in the research area of entrepreneurship.

Regarding H2, results indicate that cognitive flexibility positively influenced entrepreneurial alertness, and the hypothesis was supported. Research on cognitive flexibility is a part of cognitive psychology [113] which indicated that individuals must use an easy plan for exploring and processing information cognitively. Individuals with cognitive flexibility have more awareness and knowledge related to opportunity identification and its exploitation. Therefore, individuals with a high level of alertness and cognitive abilities are more inclined to handle uncertain environmental situations and recognize opportunities where non-entrepreneurs failed. According to Shepherd and Patzelt [24], cognitive flexibility positively influences individual cognitive abilities to identify and recognize business opportunities. Therefore, our results are similar to prior studies [23, 54], which found that individuals with cognitive abilities are more alert to identify and exploit new business opportunities in the market.

Concerning H3, findings suggest that entrepreneurial alertness has a positive impact on entrepreneurial intention, which supports the acceptance of the hypothesis. Entrepreneurial alertness enhances the individual level of searching and scanning, collecting appropriate information, and judgment of opportunity identification, which helps them to form an entrepreneurial intention and behavior. From the global perspective, numerous studies have confirmed that entrepreneurial alertness is an essential predictor for individuals because it directly influences opportunity identification and recognition [4, 23, 28, 68]. Urban [114] found that entrepreneurial alertness and entrepreneurial self-efficacy are essential cognitive predictors of exploring entrepreneurial intention. Thus, our results are similar to prior studies, which reported that entrepreneurial alertness is a significant predictor of entrepreneurial intention in Western and Asian entrepreneurial culture [25, 27, 29].

Concerning H4, we found that entrepreneurial self-efficacy strengthens the direct relationship between cognitive flexibility and entrepreneurial intention in the way that the relationship is healthier for the individuals who are higher in entrepreneurial self-efficacy than for those who are lower in entrepreneurial self-efficacy. This hypothesis was accepted. Tsai, Chang [76] found that a high level of entrepreneurial self-efficacy strengthens the association between opportunity recognition and entrepreneurial intention. Bueckmann-Diegoli and Gutiérrez [27] interpreted that entrepreneurial alertness is considered as a significant predictor to identify the opportunity and take necessary action to exploit that opportunity. Additionally, this study’s findings are in line with previous researchers who found that entrepreneurial self-efficacy as a mediator and moderator influences an individual’s beliefs to become entrepreneurs [114, 115].

Concerning H5, we found that entrepreneurial self-efficacy moderates the mediated relationship between cognitive flexibility and entrepreneurial intention by entrepreneurial alertness. This mediated relationship is not stronger for those who are higher in entrepreneurial self-efficacy, but it is reciprocal. This hypothesis is also supported. Prior researchers suggested that individuals who are lacking with self-efficacy are less motivated to achieve desired goals rather than those who have a greater level of self-efficacy [78, 82]. Newman, Obschonka [90] found that individuals who perceive a higher level of entrepreneurial self-efficacy show superior persistence and commitment toward entrepreneurship. Wilson, Kickul [84] argued that entrepreneurial self-efficacy develops a cognitive ability among individuals to accomplish tasks over others. Therefore, individuals with higher cognitive ability do not focus on the rigid compartmentalization of awareness. They reflect that different features of consciousness might be incorporated to generate unique ideas, beliefs, and actions. Thus, cognitive flexibility enables the creation of new thoughts by the integration of knowledge belonging to different cultural and social areas.

### Theoretical and practical implications

This study offers some theoretical contributions in the field of entrepreneurship and cognitive psychology. This study contributes a significant role of person-environment theory and theory of planned behavior in the context of cognitive flexibility and entrepreneurial intention. Prior studies argued that person-environment fit theory and theory of planned behavior contribute to the cognitive perspective in the domain of entrepreneurship [8, 35, 116]. Based on these theories, individuals with a high level of cognitive flexibility perceive the top fit toward a career in entrepreneurship and more likely to develop entrepreneurial intention rather than other individuals with low cognitive flexibility. This study’s findings extend the cognitive perspective in entrepreneurship research by investigating the cognitive flexibility with the mediating role of entrepreneurial alertness and the moderating effect of entrepreneurial self-efficacy in explaining an individual’s intention to become entrepreneurs in the context of Pakistani entrepreneurial culture.

Moreover, this study provides some practical implications for the researcher, health professionals, educationists, and policymakers who are directly and indirectly involved in enhancing the growth of entrepreneurship in the health sector. Firstly, the educators should pay more attention to the student’s cognitive abilities and encourage them to pursue a career in entrepreneurship. They must offer business start-up training programs for individuals from professional career development institutions or business incubation managers to develop their entrepreneurial minds. Secondly, educators must focus on students who have the cognitive abilities to become entrepreneurs and emerging the cognitive skills that facilitate them to see entrepreneurship as the right path to utilize their cognitive minds. Educators and policymakers facilitate students to pursue an entrepreneurial career as the first choice rather than to become an employee in a company. Thirdly, students should be motivated by the educators by restoring the existing structure of the curriculum regarding cognitive psychology and entrepreneurship. Entrepreneurship researchers and educators should develop some effective drivers to improve the development of cognition and promoting the business economy from the starting of new business ventures. Lastly, educators encourage and promote the drive of entrepreneurship among the students, arrange some industry visits to learn practical knowledge through interactive meetings with young and passionate entrepreneurs.

### Limitations and future directions

This study has some limitations, which suggest studies for future research directions. Firstly, we examined the role of cognitive flexibility on entrepreneurial intention and found that individuals with a higher level of cognitive flexibility are more committed to starting a new business venture. This study only focused on the entrepreneurial intention and entrepreneurial alertness of medical students rather than their actual behaviors. A future researcher can take the practical action of individuals with risk-taking as a predictor to add more contribution in the field of cognitive psychology and entrepreneurship. Secondly, the nature of our study was cross-sectional and based on a self-report questionnaire. Prior researchers [8, 117] argued that the use of a self-report questionnaire to measure cognitive flexibility and entrepreneurial intention is common in entrepreneurship research as well as this scale was used and validated by prior researchers [10, 48]. Therefore, a future study was conducted with longitudinal data using other cognitive psychology traits such as EEG, neurology imaging, and brain scanning of individuals to predict better cognitive abilities and entrepreneurial intentions. Thirdly, our study focused on public and private sector medical university students of Pakistan using a small sample size. Future researchers may also employ these constructs on different samples, e.g., SME sector entrepreneurs, to enhance their firm performance with cognitive flexibility.

## Supporting information

S1 Appendix (DOCX)

S1 Dataset (CSV)
