# Correction: Linking cognitive flexibility to entrepreneurial alertness and entrepreneurial intention among medical students with the moderating role of entrepreneurial self-efficacy: A second-order moderated mediation model

**DOI:** 10.1371/journal.pone.0259491

**Published:** 2021-10-28

**Authors:** Wang Jiatong, Majid Murad, Cai Li, Shabeeb Ahmad Gill, Sheikh Farhan Ashraf

The affiliation for the first author is incorrect. The correct affiliation is not indicated. Wang Jiatong is not affiliated with #1 but with: Postdoctoral College of Teacher Education, Zhejiang Normal University, China.
