# Supplementary material for: Linking cognitive flexibility to entrepreneurial alertness and entrepreneurial intention among medical students with the moderating role of entrepreneurial self-efficacy: A second-order moderated mediation model
Source: PLoS One. 2021 Sep 24;16(9):e0256420. doi: 10.1371/journal.pone.0256420 (PMC8462674; doi:10.1371/journal.pone.0256420)
Supplement: S1 Appendix — (DOCX) [file pone.0256420.s001.docx]

**Questionnaire**

**Cognitive Flexibility and Entrepreneurial Intention**

This research is to study the influence of cognitive flexibility on entrepreneurial intention with the mediating role of entrepreneurial alertness and moderating effect of entrepreneurial self-efficacy. This research is purely an academic purpose and not use for any other mean. Please provide the write answer. Confidentiality is highly assured, so kindly spend some of your time to respond the following questions.

**Section 1 - Consent**

I hereby fill this survey and give my consent to use the information for this study (kept confidential).

|  | Yes |  | No |
| --- | --- | --- | --- |

**Section 2 – Personal Credentials**

(Your personal credentials will be kept fully confidential and used for this research only)

***Gender***

|  | Male |  | Female |
| --- | --- | --- | --- |

***Age***

|  | 18-25 Years |  | 26-35 Years |  | 36-45 Years |  | 46-Above Years |
| --- | --- | --- | --- | --- | --- | --- | --- |

***Entrepreneurship Education***

|  | Yes |  | No |
| --- | --- | --- | --- |

***Family Business***

|  | Yes |  | No |
| --- | --- | --- | --- |

**Section 3 – Cognitive Flexibility**

Please tick the appropriate answers:

(1= Strongly Agree, 2= Agree, 3= Neutral, 4= Disagree, 5= Strongly Disagree)

| **Descriptions** | **1** | **2** | **3** | **4** | **5** |
| --- | --- | --- | --- | --- | --- |
| I can communicate an idea in many different ways. |  |  |  |  |  |
| I avoid new and unusual situations. |  |  |  |  |  |
| I feel like I never get to make decisions. |  |  |  |  |  |
| I can find workable solutions to seemingly unsolvable problems. |  |  |  |  |  |
| I seldom have choices when deciding how to behave. |  |  |  |  |  |
| I am willing to work at creative solutions to problems. |  |  |  |  |  |
| In any given situation, I am able to act appropriately. |  |  |  |  |  |
| My behavior is a result of conscious decisions that I make. |  |  |  |  |  |
| I have many possible ways of behaving in any given situation. |  |  |  |  |  |
| I have difficulty using my knowledge on a given topic in real life situations. |  |  |  |  |  |
| I am willing to listen and consider alternatives for handling a problem. |  |  |  |  |  |
| I have the self-confidence necessary to try different ways of behaving. |  |  |  |  |  |

**Section 4 – Entrepreneurial Alertness**

Please tick the appropriate answers:

(1= Strongly Agree, 2= Agree, 3= Neutral, 4= Disagree, 5= Strongly Disagree)

| **Descriptions** | 1 | 2 | 3 | 4 | 5 |
| --- | --- | --- | --- | --- | --- |
| ***Scanning and Search*** | | | | | |
| I have frequent interactions with others to acquire new information. |  |  |  |  |  |
| I always keep an eye out for new business ideas when looking for information. |  |  |  |  |  |
| I read news, magazines, or trade publications regularly to acquire new information. |  |  |  |  |  |
| I browse the internet every day. |  |  |  |  |  |
| I am an avid information seeker. |  |  |  |  |  |
| ***Association and Connection*** | | | | | |
| I see links between seemingly unrelated pieces of information. |  |  |  |  |  |
| I am good at “connecting dots”. |  |  |  |  |  |
| I often see connections between previously unconnected domains of information. |  |  |  |  |  |
| **Evaluation and Judgment** | | | | | |
| I have a gut feeling for potential opportunities. |  |  |  |  |  |
| I can distinguish between profitable opportunities and not-so- profitable opportunities. |  |  |  |  |  |
| I have a knack for telling high-value opportunities apart from low-value opportunities. |  |  |  |  |  |
| When facing multiple opportunities, I am able to select the good ones. |  |  |  |  |  |

**Section 5 – Entrepreneurial Self-Efficacy**

Please tick the appropriate answers:

(1= Strongly Agree, 2= Agree, 3= Neutral, 4= Disagree, 5= Strongly Disagree)

| **Descriptions** | 1 | 2 | 3 | 4 | 5 |
| --- | --- | --- | --- | --- | --- |
| I am convinced that I can successfully create new products. |  |  |  |  |  |
| I am convinced that I can think creatively. |  |  |  |  |  |
| I am convinced that I can successfully commercialize ideas. |  |  |  |  |  |
| I am convinced that I can successfully discover new business opportunities. |  |  |  |  |  |

**Section 6 – Entrepreneurial Intention**

Please tick the appropriate answers:

(1= Strongly Agree, 2= Agree, 3= Neutral, 4= Disagree, 5= Strongly Disagree)

| **Descriptions** | 1 | 2 | 3 | 4 | 5 |
| --- | --- | --- | --- | --- | --- |
| I am ready to do anything to be an entrepreneur. |  |  |  |  |  |
| My professional goal is to become an entrepreneur. |  |  |  |  |  |
| I will make every effort to start and run my own firm. |  |  |  |  |  |
| I am determined to create a firm in the future. |  |  |  |  |  |
| I have the firm intention to start a firm someday. |  |  |  |  |  |
| I have a strong intention to start a business someday. |  |  |  |  |  |

**Thank you**
